# Photodetection probability in quantum systems with arbitrarily strong light-matter interaction

**DOI:** 10.1038/s41598-018-36056-1

**Published:** 2018-12-13

**Authors:** Omar Di Stefano, Anton Frisk Kockum, Alessandro Ridolfo, Salvatore Savasta, Franco Nori

**Affiliations:** 1 Theoretical Quantum Physics Laboratory, RIKEN Cluster for Pioneering Research, Wako-shi, Saitama, 351-0198 Japan; 2 Wallenberg Centre for Quantum Technology, Department of Microtechnology and Nanoscience, Chalmers University of Technology, 412 96 Gothenburg, Sweden; 3 MIFT - Dipartimento di Scienze Matematiche e Informatiche Scienze Fisiche e Scienze della Terra, Università di Messina, I-98166 Messina, Italy; 4 Physics Department, The University of Michigan, Ann Arbor, Michigan 48109-1040 USA

## Abstract

Cavity-QED systems have recently reached a regime where the light-matter interaction strength amounts to a non-negligible fraction of the resonance frequencies of the bare subsystems. In this regime, it is known that the usual normal-order correlation functions for the cavity-photon operators fail to describe both the rate and the statistics of emitted photons. Following Glauber’s original approach, we derive a simple and general quantum theory of photodetection, valid for arbitrary light-matter interaction strengths. Our derivation uses Fermi’s golden rule, together with an expansion of system operators in the eigenbasis of the interacting light-matter system, to arrive at the correct photodetection probabilities. We consider both narrow- and wide-band photodetectors. Our description is also valid for point-like detectors placed inside the optical cavity. As an application, we propose a gedanken experiment confirming the virtual nature of the bare excitations that enrich the ground state of the quantum Rabi model.

## Introduction

The problem of the theoretical description of the photon-detection process was addressed by Glauber in ref.^1^. In this pioneering work, he formulated the quantum theory of photodetection and optical coherence. This theory is central to all of quantum optics and has occupied a key role in understanding light-matter interactions. In order to discuss measurements of the intensity of light, Glauber described the photon detector as a system that functions by absorbing quanta and registering each such absorption process, e.g., by the detection of an emitted photoelectron. In particular, Glauber defined an ideal photon detector as “a system of negligible size (e.g., of atomic or subatomic dimensions) which has a frequency-independent photoabsorption probability”. Since the photoabsorption is independent of frequency, such an ideal, small detector, situated at the point **r**, can be regarded as probing the field at a well defined time *t*. Glauber showed that the rate at which the detector records photons is proportional to $\langle i|{\hat{E}}^{-}({\bf{r}},t){\hat{E}}^{+}({\bf{r}},t)|i\rangle$, where |*i*〉 describes the initial state of the electromagnetic field. The operators ${\hat{E}}^{\pm }({\bf{r}},t)$ are the positive- and negative-frequency components of the electromagnetic field operator $\hat{E}({\bf{r}},t)={\hat{E}}^{+}({\bf{r}},t)+{\hat{E}}^{-}({\bf{r}},t)$, i.e., the components with terms varying as *e*^−*iωt*^ for all *ω* > 0 (positive-frequency components) or as *e*^*iωt*^ for all *ω* > 0 (negative-frequency components).

In cavity quantum electrodynamics (cavity QED)^2,3^, where atoms interact with discrete electromagnetic field modes confined in a cavity, it is often the photons leaking out from the cavity that are detected in experiments. To describe the dynamics of the atoms and the photons in the cavity, it is common to adopt a master-equation approach (see, e.g., refs^4,5^). In this approach, the field modes outside the cavity are treated as a heat bath, whose degrees of freedom are traced out when deriving the master equation. As a consequence, the master equation cannot be directly applied to derive the output field that is to be detected.

This gap between the quantum system and the external detector is typically bridged by input-output theory^6,7^, which can be used to determine the effect of the intra-cavity dynamics on the quantum statistics of the output field in a very clear and simple way. In particular, if we limit the discussion to a single cavity mode, with annihilation operator $\hat{a}$, interacting with an external field and apply the rotating-wave approximation (RWA), it is possible to obtain the output field operator ${\hat{a}}_{{\rm{out}}}(t)$ as a function of the intra-cavity field $\hat{a}(t)$ and the input field ${\hat{a}}_{{\rm{in}}}(t)$ operators^6–8^:

1 $${\hat{a}}_{{\rm{out}}}(t)={\hat{a}}_{{\rm{in}}}(t)+\sqrt{\kappa }\hat{a}(t),$$

where *κ* is an input-output coupling coefficient describing the cavity loss rate. Input-output relationships can also be obtained for more general finite-size media^9–12^.

In recent years, cavity QED has thrived thanks to an increase in the ability to control light-matter interaction at the quantum level. In particular, owing to the the advances in the detection, generation and emission of photons^13–17^, quantum systems are increasingly addressed at the single-photon level. As a consequence, there is a pressing need for a critical analysis of the applicability of the theory of photodetection^18–20^. Moreover, photon correlations are now routinely measured in the laboratory and many experiments, ranging from studying effects of strong and ultrastrong light-matter coupling to performing quantum state tomography or monitoring single-photon emitters (see, e.g.^17,21–31^), have shown their power in characterizing quantum systems. In addition, photodetection is also used for quantum-state engineering^32^ and quantum information protocols^13,33^.

For these complex systems, i.e., realistic atom-cavity systems, the theory of photodetection must be applied with great care because the light-matter interaction may modify the properties of the bare excitations in the system. If the physical excitations in such systems are superpositions of light and matter excitations, it is not immediately clear what really is measured in a photodetection experiment.

More specifically, we observe that the interaction Hamiltonian of a realistic atom-cavity system contains so-called counter-rotating terms, which allow simultaneous creation or annihilation of excitations in both atom and cavity modes (see, e.g., ref.^34^). These terms can be safely neglected through the RWA for small atom-cavity coupling rates *g*. However, when *g* becomes comparable to the resonance frequencies of the atoms or the cavity, the counter-rotating terms manifest, giving rise to a host of interesting effects^17,19,34–52^. This ultrastrong coupling (USC) regime is difficult to reach in optical cavity QED, but was recently realized in a variety of solid-state quantum systems^17,53–62^. The USC regime is challenging from a theoretical point of view because the total number of excitations in the cavity-emitter system is not conserved (only the parity of the number of excitations is)^38,63^.

In the USC regime, it has been shown that the quantum-optical master equation fails to provide the correct description of the system’s interaction with reservoirs^39,64^. It was also found^19^ that a naive application of the standard descriptions of photodetection and dissipation fail for thermal emission from a cavity-QED in the USC regime. In addition, quantum-optical normal-order correlation functions fail to describe photodetection experiments for such systems^35^. To understand why an incautious application of Glauber’s idea of photodetection together with standard input-output theory will give incorrect results, consider a USC system in its ground state |*G*〉. Due to the influence of the counter-rotating terms in the Hamiltonian, $\langle G|{\hat{a}}^{\dagger }\hat{a}|G\rangle \ne 0$, and since standard input-output theory predicts that $\langle {\hat{a}}_{{\rm{out}}}^{\dagger }\,{\hat{a}}_{{\rm{out}}}\rangle \propto \langle {\hat{a}}^{\dagger }\hat{a}\rangle$, this would imply that photons could be emitted from the ground state and then detected, which is unphysical. However, with a proper generalization of input-output theory^65^, Glauber’s idea of photodetection can still be applied to the output from a USC system^19^.

In this article, we present a general and simple quantum theory of the photodetection for quantum systems with *arbitrarily strong light-matter interaction*. We show how Glauber’s original results for the quantum theory of photodetection can be applied to systems in the USC regime. In contrast to previous works (e.g., ref.^19^), our approach does *not* require the use of input-output theory and therefore applies also to more general physical situations, where it is *not* possible to measure and/or identify the output photons. For example, our approach can describe situations where photodetectors are placed inside electromagnetic resonators. Moreover, we note that in unconventional optical resonators^66^, like plasmonic nano-cavities^67,68^, the detector could be placed in the near-field of the system. In this case, the input-output theory of ref.^19^ *cannot* be applied, but the theory of the present work *can* still be used. Moreover, the input-output relations obtained in ref.^19^ relies on many assumptions about the form of the coupling between the system and the bath, and about the form of the bath. Hence, it is far from being general. By contrast, in the present work we do *not* make any assumptions about which system operator couples to the photon detector; *nor* do we limit ourselves to a particular form of the bath/detector.

In order to calculate the detection probability of the photoabsorber, we use the more general Fermi’s golden rule. As a consequence, our approach can be applied to measurements of field correlations inside an optical resonator. In such a case, it is not possible to use the input-output approach because the interaction strongly modifies the positive- and negative-frequency field components. Their explicit expressions, in fact, contain combinations of the bare creation and destruction photon operators, which cannot be treated separately as would be required in input-output theory. In addition, using the correct positive- and negative-frequency parts of the field dressed by the interaction, we are able to calculate the photodetection probabilities for both narrow- and wide-band photodetectors.

We observe that a key theoretical issue for systems in the USC regime is the distinction between bare (unobservable) excitations and physical particles that can be detected^34^. Several works^19,40,65^ have shown that the photons in the ground state are not observable, in the sense that they do not give rise to any output photons that can be observed by standard photon detection. However, other works have shown that the photons in the ground state may be indirectly detected (without being absorbed)^44,69–71^. The formalism we develop here allow us to investigate the issue of these ground-state photons more deeply than before, elucidating their virtual nature. In particular, we apply our photodetection theory to investigate the nature of photons dressing the ground state of the quantum Rabi model. As is well-known from previous work^31,45,65,72–74^, the ground state (but also the excited states^34^) of the quantum Rabi Hamiltonian contains photons that cannot be detected by photo-absorption in a setup such as the one considered in ref. ^19^, where the detector is placed outside the cavity. However, the nature of these photons has still not been fully settled in the literature and they remain a subject of great interest (see, e.g., ref. ^73^). Sometimes they are considered virtual excitations^34^, sometimes as bound photons^72^. Our calculation in Sec. IV, beyond confirming, on a general basis, that they *cannot* be observed in any ordinary photodetection experiment, clarifies that they have all the features of virtual particles: they come into existence only for very short times, compatible with the time-energy uncertainty principle.

This article is organized as follows. In Sec. II, we derive the photodetection probability for a photoabsorber coupled to a quantum system, which may have arbitrarily strong light-matter interaction. We then show how to apply this formalism to two representative systems in Sec. III. In Sec. IV, we use the results from Sec. II to analyze the nature of photonic and atomic excitations dressing the ground and excited states in a USC system. We conclude in Sec. V.

## Excitation Probability for a Photon Detector

We consider a generic quantum system with light-matter coupling. This quantum system is weakly coupled to a photo-absorber, which is modelled as a quantum system with a collection of modes at zero temperature, described by the Hamiltonian (we set $\hslash =1$ throughout this article unless otherwise specified)

2 $${\hat{H}}_{d}=\sum _{n}\,{\omega }_{n}{\hat{c}}_{n}^{\dagger }{\hat{c}}_{n}.$$

The Hamiltonian $\hat{{\mathscr{V}}}$ describing the coupling between the light-matter system and the photo-absorber is

3 $$\hat{{\mathscr{V}}}=\sum _{n}\,{g}_{n}({\hat{c}}_{n}^{\dagger }+{\hat{c}}_{n})\hat{O},$$

where $\hat{O}$ is some operator of the light-matter system, ${\hat{c}}_{n}$ (${\hat{c}}_{n}^{\dagger }$) is an annihilation (creation) operator for mode *n* of the photo-absorber, and *g*_*n*_ is the strength with which this mode couples to the light-matter system. Typically, the operator $\hat{O}$ would be the operator of the intra-cavity electromagnetic field in a cavity-QED setup. However, this is not the only possibility. We could also have a situation where $\hat{O}$ is an operator belonging to the matter part of the system. As for the operators ${\hat{c}}_{n}$ and ${\hat{c}}_{n}^{\dagger }$, their form will depend on the model of the photo-absorber. If the photo-absorber is a collection of harmonic oscillators, ${\hat{c}}_{n}$ and ${\hat{c}}_{n}^{\dagger }$ are bosonic operators. If the photo-absorber is a generic multilevel quantum system, ${\hat{c}}_{n}=|n\rangle \langle 0|$, where |0〉 is the ground state and |*n*〉 the *n*th excited state.

The aim of this section is to calculate the excitation probability of the photo-absorber, which initially is in its ground state |0〉. The matrix element governing this excitation process is $\langle {F}_{\alpha }|\hat{{\mathscr{V}}}|I\rangle$, where |*I*〉 = |*E*_*i*_, 0〉 and |*F*_*α*_〉 = |*E*_*k*_, *n*〉 are the initial and final states, respectively, of the total system (generic light-matter system plus photo-absorber). Here, we denote the eigenstates of the Hamiltonian ${\hat{H}}_{{\rm{s}}}$ of the light-matter system by |*E*_*k*_〉 ($k=0,1,2,\ldots$), and the corresponding eigenvalues by *E*_*k*_, choosing the labelling of the states such that *E*_*k*_ > *E*_*j*_ for *k* > *j*. In order to calculate the excitation probability of the photo-absorber in the long-time limit, following standard photodetection theory, we apply Fermi’s golden rule. Summing over the possible final states, the resulting excitation probability per unit of time for the photo-absorber can be expressed as

4 $$\begin{array}{ccc}\frac{{\rm{d}}{W}_{i}}{{\rm{d}}t} & = & 2\pi \,\sum _{n,k}\,|\langle {E}_{k},n|\hat{{\mathscr{V}}}|{E}_{i},0\rangle {|}^{2}\delta ({\omega }_{n}+{E}_{k}-{E}_{i})\\  & = & 2\pi \,\sum _{n,k}\,{g}_{n}^{2}|\langle {E}_{k}|\hat{O}|{E}_{i}\rangle {|}^{2}\delta ({\omega }_{n}+{E}_{k}-{E}_{i}).\end{array}$$

If the photo-absorber has a continuous spectrum, the sum over absorber modes can be replaced by an integral over the corresponding frequencies (${\omega }_{n}\to \omega$):

5 $$\begin{array}{rcl}\frac{{\rm{d}}{W}_{i}}{{\rm{d}}t} & = & 2\pi \,{\int }_{0}^{\infty }\,{\rm{d}}\omega {g}^{2}(\omega )\rho (\omega )\\  &  & \times \,\sum _{k < i}\,|\langle {E}_{k}|\hat{O}|{E}_{i}\rangle {|}^{2}\delta (\omega +{E}_{k}-{E}_{i}),\end{array}$$

where *ρ*(*ω*) is the density of states of the absorber. Note that we also limited the summation to *k* < *i*, which follows from the delta-function terms since the continuous spectrum of the absorber only contains positive frequencies *ω*. Using some further manipulation based on this fact, Eq. (5) can be expressed as

6 $$\frac{{\rm{d}}{W}_{i}}{dt}={\int }_{0}^{\infty }\,{\rm{d}}\omega \,\sum _{k < i}\,\chi ({\omega }_{i,k})|\langle {E}_{k}|\hat{O}|{E}_{i}\rangle {|}^{2}\delta (\omega +{E}_{k}-{E}_{i}),$$

where *ω*_*i*,*k*_ = *E*_*i*_ − *E*_*k*_, and we defined *χ*(*ω*) = 2*πg*^2^(*ω*)*ρ*(*ω*). To further simplify Eq. (6), we define the positive-frequency operator

7 $${\hat{x}}^{+}=\sum _{j}\,\sum _{k < j}\,{x}_{kj}|{E}_{k}\rangle \langle {E}_{j}|,$$

where

8 $${x}_{kj}=\sqrt{\chi ({\omega }_{j,k})}{O}_{kj}=\sqrt{\chi ({\omega }_{j,k})}\langle {E}_{k}|\hat{O}|{E}_{j}\rangle .$$

Since in Eq. (6) *k* < *i*, we obtain

9 $$\sqrt{\chi ({\omega }_{i,k})}\langle {E}_{k}|\hat{O}|{E}_{i}\rangle =\langle {E}_{k}|{\hat{x}}^{+}|{E}_{i}\rangle .$$

Inserting Eq. (9) into Eq. (6), we obtain

10 $$\begin{array}{rcl}\frac{{\rm{d}}{W}_{i}}{{\rm{d}}t} & = & \sum _{k}\,{\int }_{0}^{\infty }\,{\rm{d}}\omega \langle {E}_{i}|{\hat{x}}^{-}|{E}_{k}\rangle \langle {E}_{k}|{\hat{x}}^{+}|{E}_{i}\rangle \delta (\omega +{E}_{k}-{E}_{i})\\  & = & \langle {E}_{i}|{\hat{x}}^{-}{\hat{x}}^{+}|{E}_{i}\rangle ,\end{array}$$

where we used the identity relation ${\sum }_{k}\,|{E}_{k}\rangle \langle {E}_{k}|=\hat{1}$.

The detector excitation rate is thus proportional to the initial-state expectation value of the Hermitian operator ${\hat{x}}^{-}{\hat{x}}^{+}$. We can extend this result to a more general situation where the initial state is mixed, described by the density matrix $\hat{\rho }={\sum }_{j}\,{P}_{j}|j\rangle \langle j|$, where *P*_*j*_ is the probability that the initial state is |*j*〉. In this case, the excitation probability rate becomes

11 $$\frac{{\rm{d}}{W}_{i}}{{\rm{d}}t}=\langle {\hat{x}}^{-}{\hat{x}}^{+}\rangle ,$$

where

12 $$\langle {\hat{x}}^{-}{\hat{x}}^{+}\rangle ={\rm{Tr}}\,[\hat{\rho }(t){\hat{x}}^{-}{\hat{x}}^{+}\mathrm{].}$$

If the frequency dependence of *χ*(*ω*) can be neglected, we can set $\chi (\omega )\equiv \chi$ and write the excitation probability rate as

13 $$\frac{{\rm{d}}{W}_{i}}{{\rm{d}}t}=\chi \langle {\hat{O}}^{-}{\hat{O}}^{+}\rangle ,$$

where $${\hat{O}}^{+}={\sum }_{i}\,{\sum }_{j < i}\,\,{O}_{ji}|{E}_{j}\rangle \langle {E}_{i}|$$.

We now consider the case of a narrow-band photodetector, which only absorbs excitations in a narrow band around a frequency *ω*_d_. Setting *ω*_*n*_ = *ω*_d_ and ${g}_{n}=g({\omega }_{d})\equiv g$ in Eq. (4), we obtain

14 $$\begin{array}{rcl}\frac{{\rm{d}}{W}_{i}}{{\rm{d}}t} & = & 2\pi \,\sum _{k}\,{g}^{2}|\langle {E}_{k}|{\hat{O}}^{+}(t)|{E}_{i}\rangle {|}^{2}\delta ({\omega }_{{\rm{d}}}+{E}_{k}-{E}_{i})\\  & = & \int \,{\rm{d}}\tau \,\sum _{k}\,{g}^{2}{O}_{ik}^{-}(t){O}_{ki}^{+}(t){e}^{i({\omega }_{{\rm{d}}}+{E}_{k}-{E}_{i})\tau },\end{array}$$

where

15 $$\begin{array}{rcl}{O}_{ki}^{\pm }(t) & = & \langle {E}_{k}|{\hat{O}}^{\pm }(t)|{E}_{i}\rangle =\langle {E}_{k}(t)|{\hat{O}}^{\pm }|{E}_{i}(t)\rangle \\  & = & {e}^{-i({E}_{i}-{E}_{k})t}\langle {E}_{k}|{\hat{O}}^{\pm }|{E}_{i}\rangle \mathrm{.}\end{array}$$

Observing that ${O}_{ki}^{-}={O}_{ik}^{+\dagger }$ and ${O}_{ki}^{+\dagger }(t+\tau )={O}_{ki}^{+\dagger }(t){e}^{-i({E}_{i}-{E}_{k})\tau }$, Eq. (14) becomes

16 $$\begin{array}{rcl}\frac{{\rm{d}}{W}_{i}}{{\rm{d}}t} & = & \int \,{\rm{d}}\tau \,\sum _{k}\,{g}^{2}{O}_{ik}^{-}(t){O}_{ki}^{+}(t){e}^{i({\omega }_{{\rm{d}}}+{E}_{k}-{E}_{i})\tau }\\  & = & {g}^{2}\,\int \,{\rm{d}}\tau \,\sum _{k}\,{O}_{ik}^{-}(t){O}_{ki}^{+}(t+\tau ){e}^{i{\omega }_{{\rm{d}}}\tau }\mathrm{.}\end{array}$$

Performing the summation over the possible final states *k*, we finally obtain

17 $$\frac{{\rm{d}}{W}_{i}}{{\rm{d}}t}={g}^{2}\,\int \,{\rm{d}}\tau {e}^{i{\omega }_{{\rm{d}}}\tau }{\langle {\hat{O}}^{-}(t){\hat{O}}^{+}(t+\tau )\rangle }_{i},$$

where ${\langle {\hat{O}}^{-}(t){\hat{O}}^{+}(t+\tau )\rangle }_{i}$ is the two-time expectation value with respect to the initial state.

## Applications

We now show how this formalism for photon detection can be applied to two typical quantum systems with light-matter interaction: cavity QED with natural atoms and superconducting circuits with artifical atoms and microwave photons. Once the interaction Hamiltonian with the correct system and photoabsorber operators has been identified, applying the results from Sec. II is straightforward.

### An atom as a detector for the electromagnetic field in a cavity

We first consider the electromagnetic field in a cavity, interacting with arbitrary strength with some quantum system, e.g., one or more natural atoms situated in the cavity. As our photoabsorber, we take an atom that is weakly coupled to the field (and not coupled at all to the quantum system that the cavity interacts with; such setups are recently being considered for probing systems in the USC regime^30,44^). The interaction Hamiltonian describing the field and the absorber can then be written as

18 $$\hat{{\mathscr{V}}}=-\,\frac{e}{m}\hat{{\bf{p}}}\cdot \hat{{\bf{A}}},$$

where $\hat{{\bf{p}}}$ is the atomic momentum operator, $\hat{{\bf{A}}}$ is the vector potential of the electromagnetic field, *e* is the charge of the electron orbiting the atom, and *m* is the mass of the electron. For the sake of simplicity, we are considering a one-electron (hydrogen-like) atom. We are adopting the Coulomb gauge and we neglected the ${\hat{A}}^{2}$ term, which is a good approximation in the weak-interaction regime (strength of the detector-field interaction much lower than the cavity frequency). Using Eq. (4), labelling the atomic eigenstates by |*n*〉 and the energies of these states by *ω*_*n*_ (we set the energy of the ground state |0〉 to zero), we obtain an expression for the atomic excitation rate:

19 $$\frac{{\rm{d}}{W}_{i}}{{\rm{d}}t}=\sum _{n,k}\,|\langle {E}_{k},n|\hat{{\mathscr{V}}}|{E}_{i},0\rangle {|}^{2}\delta ({\omega }_{n}+{E}_{k}-{E}_{i}).$$

By using the relationship $[\hat{{\bf{r}}},{\hat{H}}_{{\rm{d}}}]=i\hat{{\bf{p}}}/m$, where ${\hat{H}}_{{\rm{d}}}$ is the Hamiltonian of the photodetector in the absence of the interaction with light, we obtain

20 $$\langle {E}_{k},n|\hat{{\mathscr{V}}}|{E}_{i},\,0\rangle =i{\omega }_{n}\,{{\bf{d}}}_{n}\cdot \langle {E}_{k}|\hat{{\bf{A}}}|{E}_{i}\rangle ,$$

where ${{\bf{d}}}_{n}=e\langle n|\hat{{\bf{r}}}\mathrm{|0}\rangle$. Introducing the matrix element from Eq. (37) into Eq. (19) leads to

21 $$\frac{{\rm{d}}{W}_{i}}{{\rm{d}}t}=\sum _{n,k}\,|i{\omega }_{n}{{\bf{d}}}_{n}\cdot \langle {E}_{k}|\hat{{\bf{A}}}|{E}_{i}\rangle {|}^{2}\delta ({\omega }_{n}+{E}_{k}-{E}_{i}),$$

which, after introducing the positive-frequency electric-field operator

22 $${\hat{{\bf{E}}}}^{+}=\sum _{m}\,\sum _{j < m}\,-\,i({E}_{j}-{E}_{m}){\hat{{\bf{A}}}}_{jm}|{E}_{j}\rangle \langle {E}_{m}|,$$

using the Dirac delta function, and assuming a constant dipole moment **d**_*n*_ = **d** (wide-band detector), can be expressed as

23 $$\frac{{\rm{d}}{W}_{i}}{{\rm{d}}t}={d}_{\alpha }{d}_{\beta }\langle {\hat{E}}_{\alpha }^{-}{\hat{E}}_{\beta }^{+}\rangle ,$$

where the Greek letters indicate the cartesian components of the dipole moment and of the electric-field operator, and repeated indices are summed over. Note that Eq. (22) follows Glauber’s prescription^1^ for the positive-frequency operator. Specifically, switching to the Heisenberg picture, from the spectral decomposition in Eq. (22), it is clear that the operator ${\hat{{\bf{E}}}}^{+}$ includes only positive-frequency terms oscillating as exp[−*i*(*E*_*m*_ − *E*_*j*_)*t*], with *E*_*m*_ > *E*_*j*_.

We note again that if the strength of the coupling between the cavity field and the quantum system it interacts with (not the photoabsorber) is arbitrarily large, the positive- and negative-frequency electric-field operators appearing in the final expression for the photodetection probability in Eq. (23) may not correspond to the bare creation and annihilation operators *a* and *a*^†^ of that field. Instead, the photodetection probability is set by transitions between the eigenstates of the full system (cavity field plus the quantum system it interacts with).

### Circuit QED

As our second example, we consider a circuit-QED setup. In circuit QED, artificial atoms formed by superconducting electrical circuits incorporating Josephson junctions can be strongly coupled to *LC* and transmission-line resonators^17,28,75^. These circuits can be designed to explore new regimes of quantum optics. In particular, recent circuit-QED experiments^59,61^ hold the current record for strongest light-matter interaction, having reached not only the USC regime but also the regime of deep strong coupling, where the coupling strength exceeds the resonance frequencies of both the (artificial) atom and the electromagnetic mode(s). Circuit-QED systems are also being used to investigate virtual and real photons in other settings than ultrastrong light-matter interaction^76^, e.g., in the dynamical Casimir effect^77–80^.

**Figure 1:**
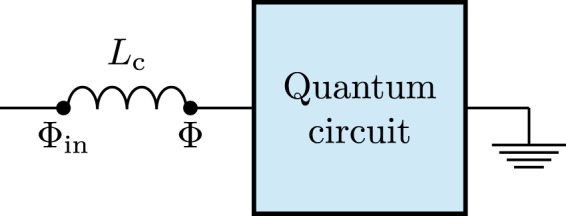
Sketch of a circuit-QED system inductively coupled to a photon detector.

As sketched in Fig. 1, we treat our (possibly quite complex) quantum circuit as a “black box”^81^. The quantum circuit will contain both electromagnetic modes and artificial atom(s), but for our purposes it is sufficient to know how this system as a whole couples to an absorbing photon detector. We assume that the coupling is through an inductor *L*_c_ that connects a node flux Φ of the circuit to a node flux Φ_in_ of the photoabsorber. Analogous results can be obtained for a quantum circuit capacitively coupled to a transmission line; see, e.g., ref.^82^.

From standard circuit quantization methods^83^, it follows that the interaction Hamiltonian for our setup is^84^

24 $${\hat{H}}_{{\rm{int}}}=\frac{1}{2{L}_{{\rm{c}}}}{(\hat{{\rm{\Phi }}}-{\hat{{\rm{\Phi }}}}_{{\rm{in}}})}^{2},$$

where the node fluxes have been promoted to quantum operators and thus acquired hats. The operator ${\hat{{\rm{\Phi }}}}_{{\rm{in}}}$ represents the measurement system that we hook up to our quantum circuit; it can be rewritten as a weighted contribution of absorber modes:

25 $${\hat{{\rm{\Phi }}}}_{{\rm{in}}}=\sum _{n}\,{k}_{n}({\hat{c}}_{n}+{\hat{c}}_{n}^{\dagger }\mathrm{).}$$

Similarly, the flux operator $\hat{{\rm{\Phi }}}$ can be expressed as^84^

26 $$\hat{{\rm{\Phi }}}=\sum _{m}\,{{\rm{\Phi }}}_{{\rm{ZPF}}}^{(m)}({\hat{a}}_{m}+{\hat{a}}_{m}^{\dagger }),$$

where ${{\rm{\Phi }}}_{{\rm{ZPF}}}^{(m)}$ is the quantum zero-point fluctuations in flux for mode *m* of the quantum circuit. Using Eqs (24–26), the interaction Hamiltonian can thus be expressed as

27 $$\begin{array}{ccc}{\hat{H}}_{{\rm{i}}{\rm{n}}{\rm{t}}} & = & \frac{1}{2{L}_{{\rm{c}}}}[\,-\,2\,\sum _{n,m}\,{k}_{n}({\hat{c}}_{n}+{\hat{c}}_{n}^{\dagger }){{\rm{\Phi }}}_{{\rm{Z}}{\rm{P}}{\rm{F}}}^{(m)}({\hat{a}}_{m}+{\hat{a}}_{m}^{\dagger })\\  &  & +\,\sum _{m,k}\,\,{{\rm{\Phi }}}_{{\rm{Z}}{\rm{P}}{\rm{F}}}^{(m)}{{\rm{\Phi }}}_{{\rm{Z}}{\rm{P}}{\rm{F}}}^{(k)}({\hat{a}}_{m}+{\hat{a}}_{m}^{\dagger })({\hat{a}}_{k}+{\hat{a}}_{k}^{\dagger })\\  &  & +\,\sum _{n,j}\,\,{k}_{n}\,{k}_{j}({\hat{c}}_{n}+{\hat{c}}_{n}^{\dagger })({\hat{c}}_{j}+{\hat{c}}_{j}^{\dagger })].\end{array}$$

Neglecting the quadratic terms in the last two lines of Eq. (27) if they can be considered small, or including them in the bare Hamiltonians of the quantum circuits and the photoabsorber, we obtain

28 $$\begin{array}{rcl}{\hat{H}}_{{\rm{int}}} & = & \frac{1}{{L}_{{\rm{c}}}}\,\sum _{n,m}\,{k}_{n}({\hat{c}}_{n}+{\hat{c}}_{n}^{\dagger }){{\rm{\Phi }}}_{{\rm{ZPF}}}^{(m)}({\hat{a}}_{m}+{\hat{a}}_{m}^{\dagger })\\  & = & \sum _{n}\,{g}_{n}({\hat{c}}_{n}+{\hat{c}}_{n}^{\dagger })\hat{O},\end{array}$$

where *g*_*n*_ = -*k*_*n*_/*L*_c_ and $$\hat{O}={\sum }_{m}\,{{\rm{\Phi }}}_{{\rm{ZPF}}}^{(m)}({\hat{a}}_{m}+{\hat{a}}_{m}^{\dagger })$$.

Observing that the operatorial form of the interaction Hamiltonian in Eq. (28) is the same as that given in Eq. (3), the results from Sec. II imply that the probability to absorb a photon from the quantum circuit in the initial state |*E*_*i*_〉 is proportional to the mean value of the operator ${\hat{x}}^{-}{\hat{x}}^{+}$, where, in this case,

29 $${\hat{x}}^{+}=\sum _{j}\,\sum _{k < j}\,\sum _{m}\,{{\rm{\Phi }}}_{{\rm{ZPF}}}^{(m)}\langle {E}_{j}|({\hat{a}}_{m}+{\hat{a}}_{m}^{\dagger })|{E}_{k}\rangle |{E}_{k}\rangle \langle {E}_{j}\mathrm{|.}$$

Of course, to find the eigenstates |*E*_*j*_〉 of the quantum circuit, a more detailed description of that system is needed. In general, these eigenstates will include contributions from both artificial atoms and resonator modes in the circuit. Thus, the operators ${\hat{x}}^{+}$ and ${\hat{x}}^{-}$ may not correspond to the bare creation and annihilation operators ${\hat{a}}_{m}$ and ${\hat{a}}_{m}^{\dagger }$.

At optical frequencies, the radiation produced by a source is frequently characterized by analysing the temporal correlations of emitted photons using single-photon counters. At microwave frequencies, however, it is difficult to develop efficient single-photon counters^16,17^. In this spectral range, signals are generally measured by using homodyne or heterodyne linear detectors. The analysis of measurements on circuit-QED systems in the USC regime requires a description of output field-quadrature measurements beyond the standard approach^31^. However, in the last years, methods able to measure normal-order correlation functions, like those considered in standard photodetection, have been developed^26,85^. The results presented here also apply when these detection methods are employed. Specifically, the method developed in ref.^26^ is based on heterodyne detection able to extract the complex envelope of the positive-frequency amplified electric field (∝${\hat{x}}^{+}$). In ref.^85^, each signal photon deterministically excites a qubit coupled to a resonator. The subsequent dispersive readout of the qubit produces a discrete “click”. This method can be regarded as a standard photoabsorption process, and the Fermi golden rule derived in Sec. II can be directly applied.

## Analysis of the Nature of Photons Dressing the Ground State of the Quantum Rabi Hamiltonian

As another application of our results from Sec. II, we now study in more detail the ground state of an ultrastrongly coupled light-matter system. We wish to clarify the question of the virtual nature of excitations in parts of the system that contribute to the ground state of the system as a whole. Using the formalism from Sec. II, we will perform a gedanken experiment which, in principle, lets us estimate the lifetime of such excitations.

### The quantum Rabi model

We consider the quantum version of the Rabi model^86^, which describes a two-level atom interacting with a single electromagnetic mode. The full system Hamiltonian is

30 $${\hat{H}}_{{\rm{R}}}={\omega }_{0}{\hat{a}}^{\dagger }\hat{a}+\frac{{\omega }_{{\rm{a}}}}{2}{\hat{\sigma }}_{z}+g{\hat{\sigma }}_{x}(\hat{a}+{\hat{a}}^{\dagger }),$$

where *a* (*a*^†^) is the annihilation (creation) operator for the electromagnetic mode, *ω*_0_ is the resonance frequency of said mode, *ω*_a_ is the transition frequency of the two-level atom, ${\hat{\sigma }}_{z}$ and ${\hat{\sigma }}_{x}$ are Pauli matrices, and *g* is the strength of the light-matter coupling.

If the coupling strength *g* is much smaller than the resonance frequencies *ω*_0_ and *ω*_a_, the RWA can be applied to reduce ${\hat{H}}_{{\rm{R}}}$ to the Jaynes–Cummings (JC) Hamiltonian^87^

31 $${\hat{H}}_{{\rm{JC}}}={\omega }_{0}{\hat{a}}^{\dagger }\hat{a}+\frac{{\omega }_{{\rm{a}}}}{2}{\hat{\sigma }}_{z}+g({\hat{\sigma }}_{-}{\hat{a}}^{\dagger }+{\hat{\sigma }}_{+}\hat{a}),$$

where ${\hat{\sigma }}_{-}$ (${\hat{\sigma }}_{+}$) is the lowering (raising) operator of the atom. The JC Hamiltonian is easy to diagonalize and has the ground state |*g*, 0〉, where |*g*〉 is the ground state of the atom and the second number in the ket indicates the number of photons in the electromagnetic mode.

However, if the coupling strength increases, the full quantum Rabi Hamiltonian must be used. This Hamiltonian can also be solved^63^; the eigenstates can be written in the form

32 $$|{E}_{j}\rangle =\sum _{k=0}^{\infty }\,({c}_{g,k}^{j}|g,k\rangle +{d}_{e,k}^{j}|e,k\rangle ),$$

where |*e*〉 denotes the excited state of the atom. In particular, the ground state of ${\hat{H}}_{{\rm{R}}}$ is

33 $$|{E}_{0}\rangle =\sum _{k=0}^{\infty }\,({c}_{g\mathrm{,2}k}^{0}|g,\,2k\rangle +{d}_{e\mathrm{,2}k+1}^{0}|e,\,2k+1\rangle )$$

with non-zero coefficients ${c}_{g,k}^{0}$ and ${d}_{e,k}^{0}$ for states that contain an even number of bare atomic and photonic excitations. Thus, if we calculate the expectation value of the bare photon number, the result is

34 $$\langle {E}_{0}|{\hat{a}}^{\dagger }\hat{a}|{E}_{0}\rangle =\sum _{k=0}^{\infty }\,(2k|{c}_{g,2k}^{0}{|}^{2}+(2k+1)|{d}_{e,2k+1}^{0}{|}^{2})\ne 0.$$

As mentioned in the introduction, several theoretical studies^19,30,31,34,40,65^ have shown that these photons that are present in the ground state cannot be observed outside the system, since they do not correspond to output photons that can be detected. The diagrammatic approach to the quantum Rabi model in ref. ^34^ also suggests that these photons should be thought of as virtual. However, there are theoretical proposals^44,69,70^ for indirect, non-demolition detection of the photons in the ground state. The question may thus arise whether these photons should be termed virtual or real.

**Figure 2:**
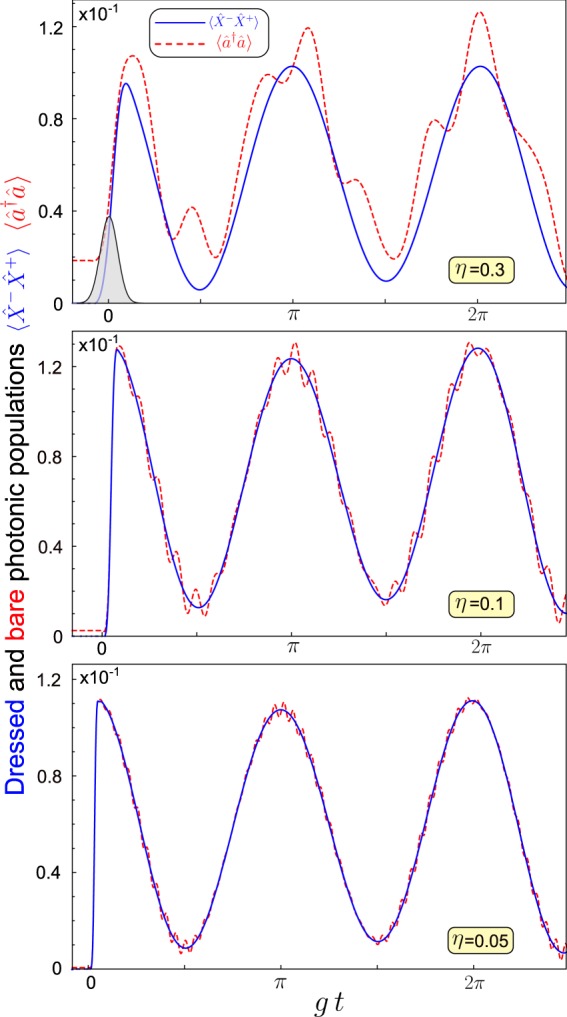
Vacuum Rabi oscillatons: time evolution of the dressed (blue continuous line) and bare (dotted line) populations for a quantum Rabi system excited by a resonant pulse. Calculations are shown for three different normalized couplings: *η* ≡ *g*/*ω*_0_ = 0.3, *η* = 0.1, and *η* = 0.05. As expected, when coupling increases, the difference between the two calculated populations becomes more evident.

We now compare the results obtained by using the standard photodetection theory with those obtained using our approach. We consider a system described by the quantum Rabi Hamiltonian in Eq. (34) and study the vacuum Rabi oscillations. Specifically, we begin with the system in its ground state. Then, we assume that the system is excited by a resonant optical pulse driving the resonator, described by the time-dependent Hamiltonian

35 $${\hat{V}}_{d}={\mathscr{A}}{\mathscr{G}}(t-{t}_{0})\,\cos \,[{\omega }_{d}(t-{t}_{0})]\,(\hat{a}+{\hat{a}}^{\dagger }),$$

where ${\mathscr{G}}$ describes a normalized Gaussian pulse arriving at time *t* = *t*_0_ with variance *σ*^2^, and ${\mathscr{A}}$ is the effective amplitude. We consider a central frequency *ω*_*d*_ = (*E*_1_ + *E*_2_)/2 − *E*_0_, and analyse the resonant case: *ω*_*a*_ = *ω*_0_. Figure 2 displays the time evolution of the dressed $\langle {\hat{X}}^{-}{\hat{X}}^{+}\rangle$, and bare $\langle {\hat{a}}^{\dagger }\hat{a}\rangle$ photonic populations, using three different values of the normalized coupling strength $\eta \equiv g/{\omega }_{0}$. For reference, the upper panel also shows the Gaussian pulse ${\mathscr{G}}(t)$ (note that its height has been arbitrary scaled).

When the normalized coupling strength is much lower than 1 (*η* = 0.05), the difference between the two quantities is very small. We can notice in the time evolution of $\langle {\hat{a}}^{\dagger }\hat{a}\rangle$ some small fast oscillations superimposed on the main signal, which is absent in $\langle {\hat{X}}^{-}{\hat{X}}^{+}\rangle$. When increasing the coupling (*η* = 0.1), the differences become more pronounced. It is also possible to observe that $\langle {\hat{a}}^{\dagger }\hat{a}\rangle$ is different from zero even before the pulse arrival. Still increasing the coupling (*η* = 0.3), the differences become drastic.

### Attempting to detect ground-state photons through absorption

Our approach to photon detection allows us to elucidate the nature of the ground-state photons in the quantum Rabi model in two ways. First, we consider whether the photons can be detected with a photo-absorber. From the treatment in Sec. II, we know that a photo-absorber coupled to the electromagnetic mode will have an excitation probability proportional to $\langle {E}_{0}|{\hat{x}}^{-}{\hat{x}}^{+}|{E}_{0}\rangle$ when the system described by the quantum Rabi model is in its ground state. Since

36 $$\begin{array}{rcl}{\hat{x}}^{+}|{E}_{0}\rangle  & = & \sum _{j}\,\sum _{k < j}\,\langle {E}_{j}|(\hat{a}+{\hat{a}}^{\dagger })|{E}_{k}\rangle |{E}_{k}\rangle \,\langle {E}_{j}|{E}_{0}\rangle \\  & = & \sum _{k < 0}\langle {E}_{0}|(\hat{a}+{\hat{a}}^{\dagger })|{E}_{k}\rangle |{E}_{k}\rangle =\mathrm{0,}\end{array}$$

because there are no terms with *k* < 0, we conclude that a photo-absorber is not able to detect any photons in |*E*_0_〉. Note that this is a more general result than what has been obtained with input-output theory. It does not only hold for photon detectors placed outside the resonator hosting the electromagnetic mode; it also holds for photon detectors placed *inside* the interacting light-matter system. This apparently trivial result confirms that ${\hat{x}}^{+}$ is a *good* annihilation-like operator for photons, in contrast to the bare photon annihilation operator $\hat{a}$, which yelds $\hat{a}|{E}_{0}\rangle \ne 0$ (when counter-rotating terms in the system Hamiltonain are included), as shown in Eq. (34).

However, it is important to point out that the presence of virtual photons in the ground state can still be indirectly probed by observing their effects on the renormalization of the energy levels^34^, or detecting the energy shifts induced on a probe qubit^44^. The latter is an example of quantum nondemolition measurement. It has also been shown that, if the parity-symmetry of an artificial atom coupled to a resonator (in the USC regime) is broken, the virtual photons in the ground state can induce parity symmetry breaking on a probe qubit weakly coupled to the resonator^69^. In each of these cases, however, no virtual photon is directly detected through photon-absorption. Finally, we observe that, in the presence of time-dependent Hamiltonians (e.g., an abrupt switch-off of the interaction)^34,35,88^, or spontaneous decay effects^40^, virtual photons can be converted (not directly detected) into real ones. These considerations apply to artificial atoms coupled to resonators, and, in general, to any (natural or artificial) two-level system coupled to any bosonic mode.

### Probability of photoabsorption for short times

The above would seem to further strengthen the case for calling the ground-state photons virtual, but another objection to that would be that virtual particles only exist for very short times, while the excitations considered here are always present in |*E*_0_〉. As a further application of our approach to photon detection, we therefore calculate the lifetime of the excitations present in the ground state. We begin by noting that the photon-detection theory used here is based on Fermi’s golden rule, and as such it gives the probability of photoabsorption for long times. We now extend this theory to short times.

Applying standard first-order perturbation theory and using Eq. (3), we can calculate the probability that a photon disappears from the state |*E*_0_〉 and one of the absorber modes is excited. Assuming $\hat{O}=\hat{a}+{\hat{a}}^{\dagger }$, the matrix element describing this process is

37 $${W}_{k,0}^{(n)}={g}_{n}\langle {E}_{k},{1}_{n}|{\hat{c}}_{n}^{\dagger }(\hat{a}+{\hat{a}}^{\dagger })|{E}_{0},0\rangle ,$$

where the second label in the kets indicates the absorber state. Due to the presence in |*E*_0_〉 of states with a non-zero number of photons, this transition matrix element is non-zero. It is interesting to observe that this matrix element would be zero for the JC model, i.e., replacing |*E*_0_〉 with |*g*, 0〉. The resulting transition probability in the case of the quantum Rabi Hamiltonian is^89^

38 $$P=\sum _{n,k}\,|{W}_{k,0}^{(n)}{|}^{2}{F}^{2}(t,{\omega }_{k,0}^{(n)}),$$

where $${\omega }_{k,0}^{(n)}={\omega }_{n}+({E}_{k}-{E}_{0})$$, and

39 $$F(t,\omega )=\frac{\sin (\omega t/2)}{\omega /2}.$$

If *t* is sufficiently large, the function *F*(*t*, *ω*) can be approximated to within a constant factor by the Dirac delta function *δ*(*ω*). In that case, we obtain that the transition rate for times $t\gg {({E}_{1}-{E}_{0})}^{-1}$

40 $$\frac{{\rm{d}}P(t)}{{\rm{d}}t}=\sum _{n,k}\,2\pi |{W}_{k,0}^{(n)}{|}^{2}\delta ({\omega }_{k,0}^{(n)}).$$

Since ${\omega }_{k,0}^{(n)} > ({E}_{1}-{E}_{0})$ is strictly larger than zero and is of the order of *ω*_0_, no transitions will be observed for large times. However, for $t\ll 1/{\omega }_{k,0}^{(n)}$,

41 $$P=\sum _{n,k}\,|{W}_{k,0}^{(n)}{|}^{2}{t}^{2},$$

and thus the photons in |*E*_0_〉 can induce transitions with a very small probability (due to the *t*^2^ term) during a small time interval. This means that the ground-state photons are coming into existence for very short times, on the order of a period of the electromagnetic mode, in agreement with the time-energy uncertainty principle. This is consistent with the interpretation of the ground-state photons as virtual rather than real. This result represents a direct manifestation of the general energy-time uncertainty principle.

It is also interesting to point out some similarity with processes in quantum field theory (QFT). One analogy consists in the non-conservation of the number of excitations. For example, in QFT, it is possible to create additional particles from the collision of two sufficiently energetic particles. Analogously, in the light-matter USC regime, if energy is conserved, one excitation can be converted into two or more excitations^43,46,51,54^. Another interesting analogy concerns virtual particles. Differently from the USC regime, in QFT the vacuum state does not explicitly contain virtual excitations. However, the interaction in the Lagrangian still allows for the creation of virtual particles from the vacuum. Specifically, the QED interaction (see, e.g.^90^)

42 $$V=-\,e\bar{\psi }{\gamma }_{\mu }{A}^{\mu }\psi $$

(*ψ* is the electron field operator, *γ*_*μ*_ are the Dirac matrices, $\bar{\psi }={\psi }^{\dagger }{\gamma }_{0}$, and *A*_*μ*_ is the vector potential) contains energy non-conserving terms describing the simultaneous excitation of one photon and one electron-positron pair (note that *ψ* contains both destruction operators for electrons and creation operators for positrons). Hence the matrix element of *V* between the vacuum state and a state with one photon and one electron-positron pair is non-zero just like the matrix element in Eq. (37). However, since the initial state (the vacuum) and the final state (one photon and one electron-positron pair) have very different energies (*ω*_*F*_ and *ω*_*I*_ respectively), owing to the energy-time uncertainty principle, these three-particle states can only exist for a very short time Δ*t*~1/(*ω*_*F*_ − *ω*_*I*_). This can be verified directly by using first-order perturbation theory as in Eq. (38). More generally, in QFT virtual particles can appear in any process described by a Feynman diagram where at some intermediate point energy conservation is violated.

## Conclusions

We have explored photon detection for quantum systems with arbitrarily strong light-matter interaction. In these systems, the very strong interaction makes light and matter hybridize such that a naive application of standard photodetection theory can lead to unphysical results, e.g., photons being emitted from the ground state of a system. While some previous works have shown how to amend input-output theory to arrive at correct expressions for the photon output flux, we have presented a more complete theory for photon detection in these systems without relying on input-output theory. We followed Glauber’s original approach for describing photon detection and found, using Fermi’s golden rule, the correct excitation probability rate for a photoabsorber interacting with the light-matter quantum system. Calculating this rate requires knowledge of the system eigenstates, such that the system operator coupling to the photoabsorber can be divided into negative- and positive-frequency components. The difference with standard photon detection arises because the strong light-matter interaction dresses the system states such that the aforementioned components no longer correspond to bare annihilation and creation operators.

We presented results for both wide-band and narrow-band photon detectors. We then showed in detail how the formalism can be applied to two representative quantum systems that can display strong light-matter interaction: cavity QED with an atom acting as the photoabsorber, and a circuit-QED setup with inductive coupling to a photon detector. Although the results we derived here were limited to second-order correlation functions, they can be directly generalized to higher-order normal-order correlation functions.

We also applied our photon-detection formalism to the quantum Rabi Hamiltonian, which describes a two-level atom interacting with a single electromagnetic mode. For large light-matter interaction, the ground state of this model contains photons, and whether these photons are virtual or real has been subject to debate. Using our formalism, we were able to clarify the nature of the ground-state photons in two ways. First, we showed that the ground-state photons, in the limit of long times, will not be detected by a photoabsorber, no matter where this photoabsorber is placed. Unlike previous results obtained with modified input-output theory, our result also holds for a detector placed inside the system (e.g., inside an optical cavity). Second, we considered a gedanken experiment, where the excitation probability of the photoabsorber is calculated for short times. We found that there is a small excitation probability for such short times, which is consistent with an interpretation where virtual photons are flitting in and out of existence on a time-scale set by the time-energy uncertainty relation. Our results thus provide further evidence for the virtual nature of the photons present in the ground state of the quantum Rabi Hamiltonian.
